# Inhibition of Rho‐kinase restores impaired relaxation of airway smooth muscle in rat pups exposed to neonatal hyperoxia

**DOI:** 10.14814/phy2.70728

**Published:** 2026-01-16

**Authors:** Ramadan B. Sopi, Qëndrim Thaçi, Thomas M. Raffay, Qëndresa Beqiraj‐Zeqiraj

**Affiliations:** ^1^ Department of Premedical Courses, Faculty of Medicine University of Prishtina Prishtina Kosovo; ^2^ Department of Pediatrics, Rainbow Babies and Children's Hospital Case Western Reserve University Cleveland Ohio USA; ^3^ Department of Pathophysiology, Faculty of Medicine University of Prishtina Prishtina Kosovo; ^4^ Pediatric Clinic University Clinical Centre of Kosovo Prishtina Kosovo

**Keywords:** bronchopulmonary dysplasia, fasudil, neonatal hyperoxia, nitric oxide, Rho‐kinase, tracheal smooth muscle, Y‐27632

## Abstract

Neonatal hyperoxia is a key contributor to bronchopulmonary dysplasia (BPD) which is characterized by airway hyperreactivity due to increased contraction and impaired relaxation of airway smooth muscle (ASM). This study investigated whether inhibition of the Rho/Rho‐kinase signaling pathway restored tracheal smooth muscle (TSM) relaxation and reactivated the nitric oxide–guanosine 3′,5′‐cyclic monophosphate (NO‐cGMP) pathway in neonatal rats exposed to hyperoxia. Newborn rats (P4) were exposed to either ambient air (AA; *n* = 61) or hyperoxia (FiO_2_ >95%; *n* = 58) for 7 days. The effects of Rho‐kinase inhibitors (Y‐27632 or fasudil) in vitro (10 μM) or in vivo (10 mg kg^−1^ day^−1^) on electric field stimulation‐induced TSM relaxation were assessed. In subsets of the experiment, tissues were pre‐incubated in a nitric oxide synthase (NOS) inhibitor—*N* ^ω^‐nitro‐L‐arginine methyl ester (L‐NAME; 100 μM) or a Rho activator—lysophosphatidic acid (LPA; 30 μM). Rho‐kinase inhibitors, both in vitro and in vivo, restored hyperoxia‐impaired TSM relaxation to levels comparable to those of ambient air TSM. The relaxant responses in tissues supplemented with Y‐27632 or fasudil were significantly increased compared to hyperoxia control (*p* < 0.01 and *p* < 0.001), and the maximal values at 20 V were 77.90 ± 3.80%; 81.20 ± 6.10% and 40.20 ± 3.60%, respectively. These Rho‐kinase inhibitor effects in TSM were attenuated by L‐NAME, indicating mechanistic action through the NO‐cGMP pathway. Activation of Rho reduced relaxation in the AA group, an effect that was reversed by Rho‐kinase inhibition. Hyperoxia impairs ASM in neonatal rats via upregulation of Rho‐kinase activity and suppression of NO‐cGMP signaling. Pharmacological inhibition of Rho‐kinase restores relaxation, highlighting its therapeutic potential for airway dysfunction in BPD.

## 1 INTRODUCTION

Neonatal airways and lungs are structurally and functionally distinct from adult lungs. In preterm infants, respiratory disorders often originate from incomplete maturation of the tracheobronchial tree (Leuthner et al., 2004). Preterm infants are at increased risk for development of obstructive lung diseases such as asthma and chronic obstructive pulmonary disease (COPD) (Anderson et al., 2023; Pulakka et al., 2023), and lower gestational age is also associated with higher wheezing risk (Chen et al., 2025; Crist & Hibbs, 2023).

Oxygen therapy is essential in managing preterm and low birth weight neonates and has increased survival in critically ill newborns; however, hyperoxia is linked to neonatal lung injury and the onset of the disease bronchopulmonary dysplasia (BPD), characterized by alveolar simplification, airway hyperactivity and pulmonary hypertension (Arjaans et al., 2018; de Wijs‐Meijler et al., 2017; Leuthner et al., 2004). BPD remains a major global health challenge (Siffel et al., 2021), thus increasing the demands for novel and effective therapies (O'Donovan & Fernandes, 2000).

Increased airway hyperactivity in childhood is strongly associated with a history of BPD and supplemental oxygen exposure, where the normal contractile and relaxant processes of airway smooth muscle (ASM) are disrupted (Dylag et al., 2020; Hack et al., 2005; Saugstad, 2003). Rodent animal models of BPD, such as newborn rats and mice exposed to hyperoxia, display characteristics of BPD including airway hyperreactivity (Raffay et al., 2016; Sopi et al., 2013). Previously, we have shown that neonatal hyperoxia decreases relaxant responses of ASM in lung parenchymal strips of rat pups due to diminished nitric oxide–guanosine 3′,5′‐cyclic monophosphate (NO‐cGMP) signaling with a corresponding increase in contraction (Ali et al., 2012; Sopi et al., 2007, 2008).

At the intracellular level, ASM contraction is controlled by myosin light chain kinase (MLCK)‐mediated phosphorylation of 20 kilo‐Dalton regulatory myosin light chain (MLC20), while MLC phosphatase (MLCP) reverses this process to promote relaxation, maintaining in this way ASM cell functional balance (Pfitzer, 2001). Rho is a monomeric G protein that activates Rho‐kinase, an enzyme which in downstream signaling phosphorylates MLCP, thereby inhibiting MLCP activity, and contributes to smooth muscle contraction via Ca^2+^ sensitization (Beqiraj‐Zeqiraj, 2022; Sanderson et al., 2008; Somlyo & Somlyo, 2000).

Smith et al. (2007), in their study demonstrated that neonatal hyperoxia increased the extent of phosphorylation of MLC_20_ and myosin‐binding subunit of smooth muscle myosin phosphatase (MYPT) tracheal strips of rat pups. Recently, we have shown in rat pups exposed to hyperoxia that airway hyperreactivity was associated with increased Rho‐kinase protein expression and enzymatic activity in lung tissue. Pharmacological inhibition of Rho‐kinase by Y‐27632 and fasudil reversed and prevented airway hyperreactivity (Beqiraj‐Zeqiraj, 2022; Beqiraj‐Zeqiraj et al., 2022). Though traditionally recognized as a central mediator of ASM contraction, growing evidence indicates that Rho/Rho‐kinase signaling is also involved in pathways that lead to smooth muscle relaxation, particularly through interactions with the NO‐cGMP axis. Rho/Rho‐kinase signaling has been shown to be involved in the downregulation of the eNOS‐NO‐cGMP signaling cascade in the pulmonary arteries of patients with COPD. Increased Rho‐kinase activity was associated with decreased eNOS expression and cGMP levels, as well as reduced endothelium‐dependent relaxation of pulmonary arteries (Bei et al., 2013). On the other hand, the NO‐cGMP signaling pathway through cGMP‐dependent protein kinase G suppresses Rho/Rho‐kinase activity (Sauzeau et al., 2000). Inhibition of NOS or stimulation of Rho by the bioactive lipid lysophosphatidic acid (LPA) increased the amplitude of contraction of pulmonary artery smooth muscle responses to contractile stimuli, while Rho‐kinase inhibition decreased the contraction of pulmonary artery smooth muscle (Boer et al., 2002).

In neonatal hyperoxia, the primary clinical challenge is often not just excessive bronchoconstriction, but a failure of the airways to adequately relax. Although the role of Rho/Rho‐kinase signaling in ASM contraction has been well characterized, its impact on relaxation and interplay with the NO‐cGMP pathway in ASM under hyperoxic conditions in neonates remains unexplored. To our knowledge, the present study is the first to investigate the effect of inhibition of Rho‐kinase on the relaxation of tracheal smooth muscle (TSM) of neonatal rats exposed to hyperoxia. Accordingly, we hypothesized that pharmacological inhibition of the Rho/Rho‐kinase pathway restores the impaired relaxation of the TSM of hyperoxia‐exposed neonatal rats through NO‐cGMP signaling.

## 2 MATERIALS AND METHODS

### 2.1 Animals and experimental design

Experiments were performed on Wistar rat pups. Pups (P4) from two different litters were randomly mixed and assigned either to hyperoxia (H; >95%O_2_) (*n* = 58; 30 males and 28 females) or ambient air (AA) (*n* = 61; 34 males and 27 females) groups for 7 days. Food (D.O.O.GEBI, Serbia; Cat. no. GRS‐30‐004) and water were available ad libitum and 12 h light/dark cycles were maintained. Hyperoxia‐exposed pups were housed with their mothers in a commercial rat cage placed into a Plexiglas box (38 L) receiving a constant oxygen supply (2 L/min). To avoid hyperoxic toxicity, mothers were rotated every 24 h between hyperoxic and ambient air groups. The oxygen concentration was monitored continuously using a MiniOX‐1 analyzer (MiniOX‐1, Ohio Medical Corporation, IL, USA). AA groups of animals were housed in standard cages under ambient air conditions. Subsets from each group were supplemented by intraperitoneal (i.p.) injection with one of two Rho‐kinase inhibitors: (+)‐(R)‐trans‐4‐(1‐aminoethyl)‐N‐(4‐pyridyl) cyclohexane carboxamide (Y‐27632; 10 mg kg^−1^ day^−1^; Tocris Biotechne, Germany; Cat. no. 1254) or fasudil (10 mg kg^−1^ day^−1^; Tocris Biotechne, Germany; Cat. no. 0541) for 7 days during hyperoxia or ambient air exposure. The doses were standardized based on our previous study and thesis work (Beqiraj‐Zeqiraj, 2022; Beqiraj‐Zeqiraj et al., 2022). Control groups received an equal volume of saline solution.

This study was conducted in compliance with the rules described in guidelines for use of laboratory animals and the protocol was approved by the ethical research committee of the Faculty of Medicine, University of Prishtina (nr. 2550/13 and 052–23,413).

### 2.2 TSM preparation

After exposure, animals were euthanized using CO_2_ asphyxiation, and the trachea was carefully excised and cleaned of surrounding connective tissues in chilled, oxygenated Krebs–Henseleit (KH) buffer solution [(mM): 118.2 NaCl, 25 NaHCO_3_, 4.6 KCl, 1.2 KH_2_PO_4_, 1.2 MgSO_4_, 2.5 CaCl_2_, and 10% D‐glucose; pH 7.4 (all reagents from Sigma‐Aldrich, Germany)]. A 3 mm segment from the mid‐trachea was isolated from each animal and placed into a 10 mL tissue‐organ bath containing KH buffer maintained at 37°C, following established protocols (Sopi et al., 2012).

### 2.3 In vitro measurement of TSM relaxation

Tracheal preparations were mounted in tissue‐organ baths by suspending them between a stainless‐steel hook at the base of the bath and a force‐displacement transducer. The responses of the tracheal smooth muscle (TSM) were measured using a four‐channel organ bath system (DMT‐750TOBS, Danish Myo Technology, Aarhus N, Denmark), connected to a Power Lab/8SP data acquisition system (AD Instruments Inc., CO, USA). Data were monitored and recorded using Chart 8.0 software interfaced with a computer. Muscle tension was expressed in grams (g). An initial load of 0.3 g was applied, then tissues were allowed to equilibrate for 45 min in organ baths containing 10 mL of KH buffer maintained at 37 C. During the equilibration period, preparations were rinsed every 15 min with fresh KH buffer and continuously aerated with a gas mixture of 95% O_2_ and 5% CO_2_.

### 2.4 Electrical field stimulation (EFS)‐induced relaxation of TSM

To evaluate the effect of hyperoxia on relaxant responses of TSM, preparations obtained from both hyperoxia‐ and ambient air‐exposed animals were placed in organ baths as described above. A cumulative concentration‐response curve to the cholinergic bronchoconstrictor bethanechol (Sigma‐Aldrich, Germany; Cat. No. C5259) was built in preparation for these experiments. A standard concentration of 100 μM bethanechol was found to optimally elicit 75% of the maximal response in test TSM preparations. Experimental TSM preparations were pre‐constricted using a single dose of bethanechol (100 μM), then incremental EFS current was applied to the pre‐constricted TSM through platinum electrodes [1–20 V alternating current (AC) at 50 Hz] for 10 s at 2‐min intervals to induce relaxation. The relaxation of the TSM was expressed as a percentage (%) of the pre‐constricted state for each preparation.

After recording the EFS‐induced relaxant responses of pre‐contracted TSM as above, the buffer was thoroughly washed out and the TSM were similarly allowed to equilibrate. The TSM preparations were next incubated in vitro with Rho kinase inhibitors, Y‐27632 (10 μM) or fasudil (10 μM), for 30 min. After incubation, TSM were similarly pre‐constricted with 100 μM bethanechol then EFS was applied to evaluate relaxation responses.

To assess the in vivo effects of Rho‐kinase inhibitors, tracheal preparations from pups treated daily with Y‐27632 or fasudil were used to evaluate the relaxation responses to EFS.

In a subset of experiments, after first recording EFS‐induced relaxation responses, tissues were pre‐incubated in vitro with a nitric oxide synthase (NOS) inhibitor– *N*ω‐nitro‐L‐arginine methyl ester (L‐NAME, 100 μM; Sigma‐Aldrich, Germany; Cat. No. N5751) for 30 min or a Rho activator– lysophosphatidic acid (LPA, 30 μM; Tocris Biotechne, Germany; Cat. No. 3854) for 5 min prior to EFS application. Preparations were co‐incubated with or without Y‐27632 (10 μM) for 30 min.

### 2.5 Statistical analysis

Data are presented as mean ± sem. Statistical significance was determined by two‐way analysis of variance (ANOVA) with repeated measurements to assess the effect of EFS on relaxation responses between hyperoxia vs. room air groups. If indicated, post hoc comparisons were performed using a Tukey–Kramer multiple comparison test. A *p* < 0.05 was considered statistically significant, in all cases.

## 3 RESULTS

### 3.1 Effect of neonatal hyperoxia on relaxation of TSM

**FIGURE 1:**
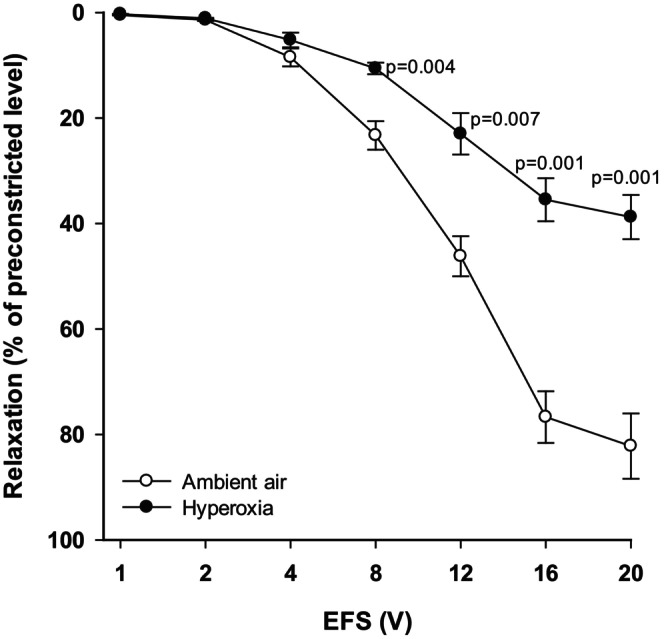
Effect of hyperoxia on EFS‐induced relaxation of the TSM of rat pups. EFS‐induced relaxation of TSM was impaired in preparation obtained from hyperoxia‐exposed rat pups (*n* = 10) as compared to those from AA‐exposed rat pups (*n* = 10). H‐hyperoxia; AA‐ambient air. Data represent mean ± sem. This graph is adapted from the doctoral thesis of the corresponding author (Beqiraj‐Zeqiraj, 2022) and the data presented here have not been published previously in any form of publication.

In hyperoxia‐exposed rat pups, relaxation of TSM induced by EFS overall was significantly reduced when compared to responses of TSM obtained from AA pups (*p* < 0.001). As shown in Figure 1, the relaxant responses of TSM from pups exposed to hyperoxia (*n* = 10) were significantly lower than those from TSM of AA pups (n = 10), particularly at higher voltages (8 – 20 V). The data of relaxant responses in hyperoxic and AA groups ranged from 0.30 ± 0.04% at 1 V to 38.80 ± 4.20% at 20 V, and from 0.50 ± 0.05% at 1 V to 82.20 ± 6.20% at 20 V, respectively. This graph is adapted from the doctoral thesis of the corresponding author (Beqiraj‐Zeqiraj, 2022).

### 3.2 In vitro and in vivo effect of Y‐27632 or fasudil on relaxation of TSM obtained from hyperoxia‐ and AA‐exposed rat pups

**FIGURE 2:**
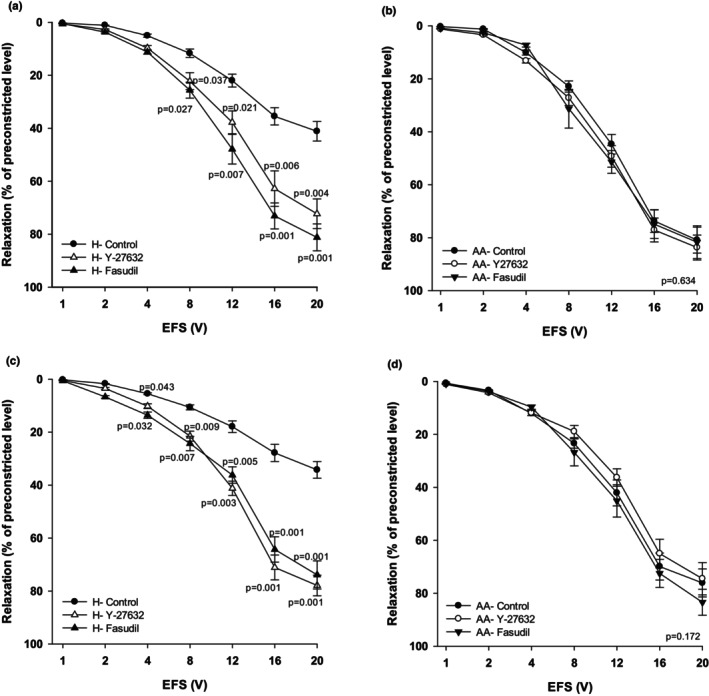
Effect of in vitro and in vivo inhibition of Rho‐kinase by Y‐27632 or fasudil on EFS‐induced relaxation of TSM of rat pups. In vitro, administered Y‐27632 (10 μM) or fasudil (10 μM) in tracheal preparations of rat pups exposed to hyperoxia‐H (panel a) or Ambient air – AA (panel b) (*n* = 10 and *n* = 12, respectively). In vivo, supplemented Y‐27632 or fasudil to the animals (10 mg kg^−1^ day^−1^) during exposure to hyperoxia (panel c) or Ambient air (panel d) (*n* = 10 per group/each condition). Each set of experiments performed in duplicate and data represent mean ± sem.

In vitro inhibition of Rho‐kinase activity reversed the effects of hyperoxia on relaxant responses of TSM. The relaxant responses of TSM in the hyperoxic group were significantly increased when the preparations were pre‐incubated in Rho‐kinase inhibitors – Y‐27632 or fasudil (overall, *p* < 0.01 and *p* < 0.001, respectively) as compared to hyperoxia control relaxant responses (Figure 2a; *n* = 10, per each condition). The relaxant responses significantly increased compared to control responses, especially at higher voltages (8–20 V). The data of relaxation of TSM incubated with Y‐27632 or fasudil ranged from 0.60 ± 0.04% at 1 V to 72.30 ± 6.60% at 20 V, and 0.50 ± 0.03% at 1 V to 81.20 ± 6.10% at 20 V, respectively. Whereas, in the hyperoxia control group the relaxant responses varied from 0.20 ± 0.03% at 1 V to 40.20 ± 3.60% at 20 V.

Y‐27632 or fasudil administered in vitro did not affect the relaxation responses of TSM obtained from the AA group of pups (Figure 2b). The data obtained from AA control, AA + Y‐27632 and AA + fasudil ranged from 0.30 ± 0.03% at 1 V to 80.90 ± 4.90% at 20 V; 0.45 ± 0.02% at 1 V to 83.69 ± 4.70% at 20 V, and 0.90 ± 0.02% at 1 V to 81.70 ± 6.20% at 20 V, respectively.

Supplementation of animals with Rho‐kinase inhibitors (Y‐27632, 10 μM or fasudil, 10 μM) during hyperoxic exposure prevented the hyperoxia‐induced impairment of TSM relaxation. Both inhibitors significantly increased (overall, *p* < 0.001) EFS‐induced relaxation of TSM of animals exposed to hyperoxia compared to saline treated H‐controls (Figure 2c).

When Y‐27632 was supplemented in hyperoxic animals the relaxant responses were significantly greater (*p* < 0.001) compared to relaxant responses from hyperoxic control animals (*n* = 10, each group) and these relaxant responses did not significantly differ from AA animals. The differences were significant at voltages 4–20 V (Figure 2c). The data obtained from the H + Y‐27632 group ranged from 0.50 ± 0.04% at 1 V to 77.90 ± 3.80% at 20 V; while in control H‐group the data varied from 0.25 ± 0.03% at 1 V to 34.25 ± 3.15% at 20 V.

Reduced relaxation due to hyperoxic exposure was also restored when fasudil was supplemented to the animals, and these relaxant responses were significantly greater (overall, *p < 0.001*) compared to relaxant responses from hyperoxic control animals (n = 10). The differences were significant at voltages 4–20 V (Figure 2c). The data obtained from H + fasudil ranged from 0.60 ± 0.03% at 1 V to 73.90 ± 5.30% at 20 V.

**FIGURE 3:**
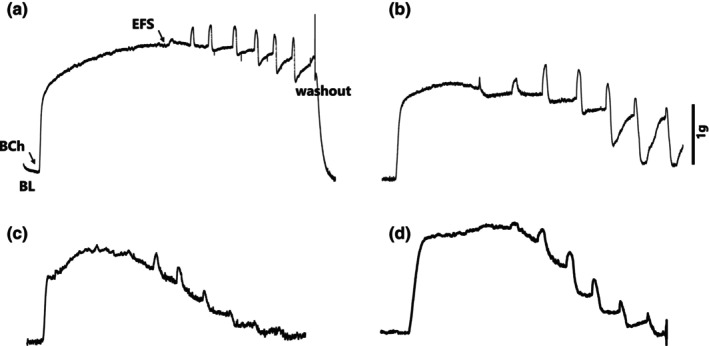
Tracings of representative recordings showing the EFS‐induced relaxation of preconstricted TSM: (a) H‐control; (b) AA‐control; (c) H‐Y27632; and (d) H‐Fasudil. BCh, Bethanechol, BL, Baseline; EFS, electrical field stimulation.

In vivo inhibition of Rho‐kinase activity by Y‐27632 or fasudil did not show any significant change in the EFS‐induced relaxation of TSM in AA‐exposed animals (*n* = 10, each group) (Figure 2d). The data obtained from AA control, AA + Y‐27632, and AA + fasudil ranged from 0.70 ± 0.04% at 1 V to 76.10 ± 5.30% at 20 V; 1.10 ± 0.01% at 1 V to 74.5 ± 6.10% at 20 V; and 0.90 ± 0.03% at 1 V to 83.40 ± 4.90% at 20 V, respectively. The tracings of representative recordings are presented in Figure 3.

### 3.3 Modulation of Rho or Rho‐kinase alters NOS‐NO‐cGMP signaling pathway in TSM

**FIGURE 4:**
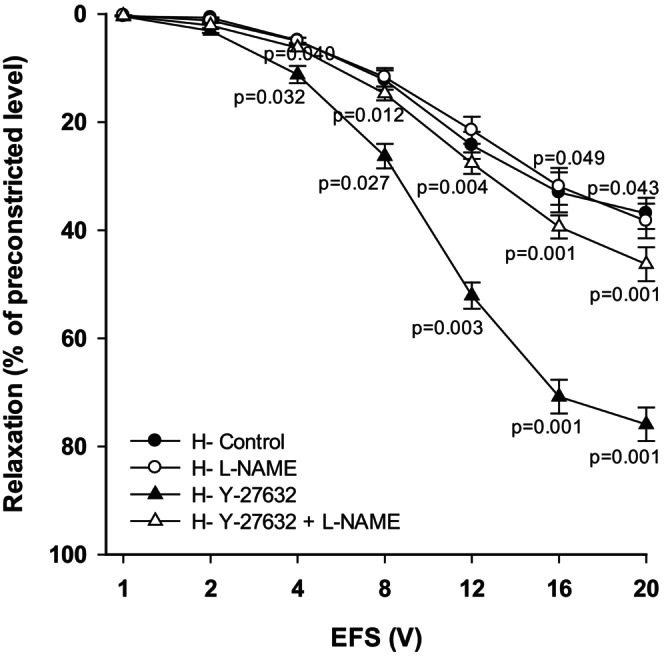
Inhibition of Rho‐kinase is modulated in part by NOS‐NO‐cGMP signaling in TSM of hyperoxia‐exposed rat pups. Incubation of hyperoxic tissues in L‐NAME (100 μM) alone did not change relaxant responses, while in the presence of Y‐27632 (10 μM), it reduced these responses (*n* = 8 each condition, and each performed in duplicate). Data represent mean ± sem.

In tracheal preparations of hyperoxic animals, inhibition of NOS with L‐NAME did not alter relaxant responses compared to controls, indicating that this signaling pathway was impaired under hyperoxic conditions. Supplementation of tissues with the Rho‐kinase inhibitor Y‐27632 restored relaxant responses, which were significantly diminished by co‐incubation of tissues with L‐NAME and Y‐27632 (overall, *p* < 0.001; Figure 4, *n* = 8). Although L‐NAME attenuated the effects of Y‐27632, the responses did not fully return to levels observed in hyperoxic controls. The differences were significant at higher voltages only, 16 V and 20 V (*p* < 0.05). The differences in relaxation responses between H + Y‐27632 and H + Y‐27632 + L‐NAME/H + L‐NAME/H‐control were significant at voltages 8 V–20 V. The data obtained from H‐control, H + L‐NAME, H + Y‐27632 and H + Y27632 + L‐NAME ranged from 0.40 ± 0.03% at 1 V to 36.90 ± 2.20% at 20 V; 0.26 ± 0.02% at 1 V to 38.30 ± 3.20% at 20 V; 0.45 ± 0.05% at 1 V to 75.92 ± 3.10% at 20 V and 0.32 ± 0.02% at 1 V to 46.30 ± 3.14% at 20 V, respectively.

**FIGURE 5:**
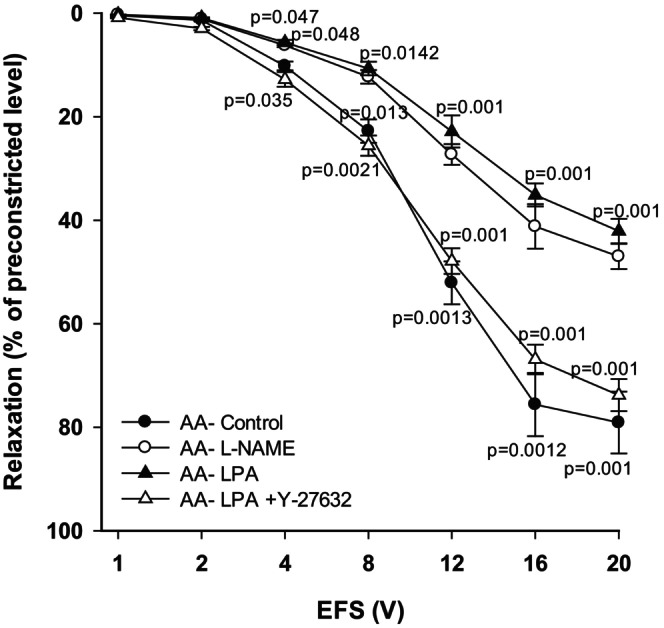
The effect of L‐NAME, LPA, and Y‐27632 on EFS‐induced relaxation of TSM of ambient air exposed rat pups. LPA (30 μM) administration significantly reduced EFS‐induced TSM relaxation compared to control responses obtained from AA rat pups. Y‐27632 (10 μM) reversed the effect of LPA and relaxant responses were restored to control level. L‐NAME (100 μM) significantly reduced relaxation of TSM (*n* = 9 each condition, and each performed in duplicate). Data represent mean ± sem.

To assess the involvement of Rho activation in hyperoxia‐impaired NOS‐NO‐cGMP signaling in tracheal preparations, we used tissues obtained from the AA group, in which this pathway remains intact. Inhibition of NOS with L‐NAME significantly reduced relaxant responses (overall, *p* < 0.001; Figure 5, *n* = 9) compared to the control responses (AA‐control). Likewise, relaxant responses were significantly reduced (overall, *p* < 0.001) following administration of the Rho activator lysophosphatidic acid (LPA; 30 μM). This LPA‐induced reduction in TSM relaxation was reversed with Rho‐kinase inhibition by Y‐27632, normalizing the relaxant responses (overall, *p* < 0.001).

The differences between AA+LPA and AA‐control or AA+LPA + Y‐27632 were significant at voltages 4 – 20 V. Also, between AA + L‐NAME and AA‐control the differences were significant at 4 – 20 V. The data obtained from AA‐control, AA+L‐NAME, AA+LPA and AA‐LPA + Y27632 ranged from 0.43 ± 0.06% at 1 V to 79.10 ± 5.98% at 20 V; 0.29 ± 0.03% at 1 V to 47.00 ± 2.93% at 20 V; 0.28 ± 0.02% at 1 V to 42.10 ± 2.37% at 20 V and 0.38 ± 0.07% at 1 V to 73.80 ± 3.10% at 20 V, respectively.

## 4 DISCUSSION

This study provides novel evidence that hyperoxia‐induced impairment of ASM relaxation in neonatal rats is mediated by upregulation of the Rho/Rho‐kinase signaling pathway, which in turn disrupts the NO‐cGMP signaling pathway. Importantly, pharmacological inhibition of Rho‐kinase with either Y‐27632 or fasudil, both in vitro and in vivo, significantly restored or protected the relaxant responses of TSM in hyperoxia‐exposed neonatal rats. These findings support our hypothesis that Rho/Rho‐kinase pathway activation contributes to the disruption of key relaxant signaling pathways in airways exposed to neonatal hyperoxia and that targeting this pathway can reverse the airway dysfunction associated with BPD.

BPD is a chronic inflammatory lung disease associated with arrested pulmonary development and a need for supplemental oxygen (Rivera et al., 2016). Prolonged hyperoxic exposure of immature lungs contributes to neonatal lung injury and is manifested by increased airway reactivity in both human infants and neonatal animal models. Such hyperoxia‐induced lung injury is characterized by both enhanced contraction and impaired relaxation of ASM in early life. In this study, exposure to hyperoxia resulted in a significant reduction in EFS‐induced TSM relaxation compared to AA controls. These findings are consistent with prior publications indicating that hyperoxic exposure alters ASM function by enhancing contractile tone and diminishing relaxation responses (Ali et al., 2012; Kryeziu et al., 2023). The Rho/Rho‐kinase signaling pathway plays an important role in airway hyperreactivity. Hyperoxic exposure upregulated both the expression and activity of Rho‐kinase in the lungs (Beqiraj‐Zeqiraj et al., 2022), which in downstream signaling targets the contractile apparatus, thus extending the contraction of smooth muscle (Yasuda et al., 2024). Inhibition of Rho‐kinase prevents the airway hyperreactivity induced by hyperoxia in rat pups (Beqiraj‐Zeqiraj et al., 2022). Inhaled Y‐27632 reversed allergen‐induced airway hyperresponsiveness after early and late asthmatic reaction in guinea pigs, confirming the involvement of Rho‐kinase in airway hyperreactivity (Schaafsma et al., 2006). Recently, a study revealed that Rho‐kinase inhibition suppresses the allergic airway hyperresponsiveness and inflammation in guinea pigs sensitized to ovalbumin (Gondáš et al., 2024).

This study confirms that hyperoxia‐induced TSM relaxation impairment is mainly controlled by the Rho/Rho‐kinase signaling pathway. Both Y‐27632 and fasudil significantly restored relaxation responses when administered in vitro. Furthermore, in vivo supplementation with Rho‐kinase inhibitors during the hyperoxic exposure prevented the decline in TSM relaxation. Interestingly, neither inhibitor altered relaxation in AA‐exposed controls, indicating that under normoxic conditions, Rho‐kinase signaling does not play a dominant role in modulating baseline airway relaxation, as noted by other investigators (Fukata et al., 2001).

We acknowledge that Y‐27632 and fasudil, although widely used as Rho‐kinase inhibitors, are not entirely selective and may exert off‐target effects, particularly at higher concentrations. It was reported for their actions on kinases such as protein kinase C (PKC) and MLCK, which can also influence smooth muscle contractility (Davies et al., 2000; Ishizaki et al., 2000). While the consistency of our in vitro and in vivo findings supports a central role for Rho‐kinase signaling in the observed effects, we recognize that contributions from non‐Rho‐kinase targets cannot be fully excluded. Future studies employing more selective pharmacologic approaches will be important to further clarify the specific role of Rho‐kinase in neonatal airway smooth muscle regulation.

Previous studies have shown that hyperoxia disturbs the key signaling pathways involved in ASM relaxation, most notably the NO‐cGMP signaling pathway (Ali et al., 2012; Sopi et al., 2007), producing a phenotype with impaired relaxation of ASM. Rho/Rho‐kinase signaling and NOS‐NO‐cGMP signaling interplay between each other. NO is synthesized from the amino acid L‐arginine by the enzyme NOS, and it activates soluble guanylyl cyclase (sGC), which produces cGMP. cGMP then activates its downstream target protein kinase G (PKG), which phosphorylates target molecules, leading to cellular responses like smooth muscle relaxation (Thoonen et al., 2013). Activation of NO‐cGMP via PKG inhibits the Rho/Rho‐kinase signaling pathway (Sauzeau et al., 2000). On the other hand, Rho/Rho‐kinase suppresses the eNOS‐NO‐cGMP signaling pathway (Bei et al., 2013). Our mechanistic experiments indicate that in neonatal hyperoxia, Rho‐kinase inhibits the NO‐cGMP signaling pathway, a prominent smooth muscle relaxation pathway. When NOS activity was inhibited by L‐NAME in hyperoxic tracheal tissues, it did not change relaxation responses, confirming that this NO pathway was functionally silenced as inhibition of Rho‐kinase only weakly restored relaxation responses. These data indicate that under hyperoxia, activation of Rho‐kinase suppresses NO production or downstream signaling, and that inhibition of this kinase can reactivate this pathway, provided evidence there is active NOS. To prove the interplay between these two signaling pathways, in the AA‐exposed group, inhibition of NOS and activation of Rho was tested. L‐NAME administered alone reduced relaxation of TSM, indicating the dominance of this pathway under normal physiological conditions. LPA activation of the Rho/Rho‐kinase pathway mimicked the effect of NOS inhibition, reducing the relaxation of TSM obtained from AA animals, and this effect of LPA was notably reversed by Rho‐inhibition. This suggests that Rho‐kinase overactivation not only promotes ASM contraction directly but also indirectly impairs relaxation via suppression of NO bioavailability or signaling. This is consistent with the described roles of Rho/Rho‐kinase in smooth muscle relaxation of vessels. Effects of NO have been shown to be counteracted by Rho‐kinase in the pulmonary artery of rats (Boer et al., 2002). Also, in vascular endothelial cells, hypoxia‐induced downregulation of eNOS expression was mediated by Rho‐kinase (Takemoto et al., 2002). Similarly, Rho‐kinase suppressed eNOS in the endothelium of the corpus cavernosum of rats (Bivalacqua et al., 2004).

Although our findings strongly support a functional interaction between Rho‐kinase activation and NO‐cGMP pathway suppression, the study did not directly measure NO levels, eNOS expression, or cGMP activity in this study, and we are aware of this limitation. As such, the mechanistic interplay we propose remains correlative. Future studies incorporating biochemical quantification of NO production, eNOS activity, and cGMP accumulation will be essential to confirm this interaction at the molecular level.

It is also important to consider that Rho‐kinase signaling may behave differently in neonatal compared to adult tissues. Belik et al. (2006) in their study revealed the changes in activity of Rho‐kinase during rat lung development where Rho‐kinase inhibition showed a higher effect in relaxation of neonatal vascular tissues than those obtained from adults. This study supports the idea that the neonatal “window” is a period of elevated ROCK‐dependent tone. These developmental differences likely help to explain why hyperoxia has such a strong effect in the neonatal model, and Rho‐kinase inhibition appears particularly effective at this stage.

## 5 CONCLUSION

These findings have important implications for neonatal airway and lung diseases such as BPD, which is characterized by disrupted alveolar and vascular development, airway hyperreactivity, and persistent pulmonary dysfunction. BPD‐affected neonates are usually treated with long‐term oxygen therapy, and airway hyperreactivity associated with impaired relaxation is a persistent consequence. Our data suggest that Rho‐kinase–mediated suppression of NO‐cGMP signaling may contribute to this phenotype and that early pharmacological inhibition of Rho‐kinase could be a novel intervention strategy to preserve or rescue airway function in at‐risk neonates. While systemic Rho‐kinase inhibition may have off‐target cardiovascular effects, local administration such as inhaled delivery could limit systemic exposure. Inhaled ROCK inhibitors have shown promise in adult pulmonary hypertension (Fujita et al., 2010), but their safety and efficacy in neonates, especially premature infants, remain to be thoroughly evaluated.

## AUTHOR CONTRIBUTIONS

RBS: conceptualization and designing the experiments, funding acquisition, performed experiments, data interpretation, drafting the manuscript; QT: formal analysis, performed experiments, reading and editing the manuscript; TMR: data interpretation, reading and editing the manuscript; QZB: conceptualization and designing the experiments, funding acquisition, performed experiments, data interpretation, review and editing the manuscript. All authors read and approved the final draft of the manuscript.

## CONFLICT OF INTEREST STATEMENT

The authors have no conflict of interest to declare.

## ETHICS STATEMENT

This study was approved by the Ethical Research Committee of the Faculty of Medicine, University of Prishtina (nr. 2550/13 and 052–23,413).

## Data Availability

The data that support the findings of this study are available from the corresponding author upon request.
